# Involvement of tumor immune microenvironment metabolic reprogramming in colorectal cancer progression, immune escape, and response to immunotherapy

**DOI:** 10.3389/fimmu.2024.1353787

**Published:** 2024-07-25

**Authors:** Andrea Nicolini, Paola Ferrari

**Affiliations:** ^1^ Department of Oncology, Transplantations and New Technologies in Medicine, University of Pisa, Pisa, Italy; ^2^ Unit of Oncology, Department of Medical and Oncological Area, Azienda Ospedaliera-Universitaria Pisana, Pisa, Italy

**Keywords:** metabolic reprogramming, colorectal cancer, tumor immune microenvironment, tumor immune escape, immunotherapy

## Abstract

Metabolic reprogramming is a k`ey hallmark of tumors, developed in response to hypoxia and nutrient deficiency during tumor progression. In both cancer and immune cells, there is a metabolic shift from oxidative phosphorylation (OXPHOS) to aerobic glycolysis, also known as the Warburg effect, which then leads to lactate acidification, increased lipid synthesis, and glutaminolysis. This reprogramming facilitates tumor immune evasion and, within the tumor microenvironment (TME), cancer and immune cells collaborate to create a suppressive tumor immune microenvironment (TIME). The growing interest in the metabolic reprogramming of the TME, particularly its significance in colorectal cancer (CRC)—one of the most prevalent cancers—has prompted us to explore this topic. CRC exhibits abnormal glycolysis, glutaminolysis, and increased lipid synthesis. Acidosis in CRC cells hampers the activity of anti-tumor immune cells and inhibits the phagocytosis of tumor-associated macrophages (TAMs), while nutrient deficiency promotes the development of regulatory T cells (Tregs) and M2-like macrophages. In CRC cells, activation of G-protein coupled receptor 81 (GPR81) signaling leads to overexpression of programmed death-ligand 1 (PD-L1) and reduces the antigen presentation capability of dendritic cells. Moreover, the genetic and epigenetic cell phenotype, along with the microbiota, significantly influence CRC metabolic reprogramming. Activating RAS mutations and overexpression of epidermal growth factor receptor (EGFR) occur in approximately 50% and 80% of patients, respectively, stimulating glycolysis and increasing levels of hypoxia-inducible factor 1 alpha (HIF-1α) and MYC proteins. Certain bacteria produce short-chain fatty acids (SCFAs), which activate CD8+ cells and genes involved in antigen processing and presentation, while other mechanisms support pro-tumor activities. The use of immune checkpoint inhibitors (ICIs) in selected CRC patients has shown promise, and the combination of these with drugs that inhibit aerobic glycolysis is currently being intensively researched to enhance the efficacy of immunotherapy.

## Introduction

Preventing cancer’s origin and progression, also known as tumor immune surveillance, is among the responsibilities of the human immune system (1, 2). However, many factors and mechanisms, primarily within the tumor microenvironment (TME), influence tumor immune surveillance (3–5), while others facilitate tumor immune escape. Tumor cells rapidly adapt to hypoxia and nutrient deficiencies that occur during tumor progression. This adaptation involves changes in the bioenergetic systems of cancer cells, termed “metabolic reprogramming,” which is one of the primary hallmarks of cancer. Metabolic reprogramming impacts cell activity and its ability to differentiate, with numerous factors interacting within the TME to account for various metabolic phenotypes. Alterations in metabolic cells within the TME can also promote tumor immune escape, where cancer and immune cells collaborate to establish a suppressive tumor immune microenvironment (TIME). The recent surge in research interest regarding metabolic TME reprogramming as a mechanism of tumor progression and a potential therapeutic target, especially in colorectal cancer (CRC)—one of the most common cancers—has prompted us to address this issue. The first part of this review highlights the common features of metabolic reprogramming in cancer and immune cells, as well as their roles in facilitating tumor immune escape. Subsequently, we provide a comprehensive overview of the current knowledge on metabolic reprogramming, immune escape, and the use of immunotherapy in CRC.

## Metabolic reprogramming in the TME

In the TME, tumors typically exhibit high blood content and oxygen consumption, while exporting lactate to the extracellular space through aerobic glycolysis. This process leads to acidification (6) and hypoxia within the TME (7). Furthermore, the interaction between cancer and immune cells contributes to their metabolic reprogramming and the shaping of the microenvironment.

### Metabolic interactions between cancer and immune cells

Within the TME, cancer cells and immune cells compete for the same limited metabolic resources. The proliferation of cancer cells is associated with increased metabolic demands, which also affect the metabolic requirements of immune cells, influencing their function and fate. However, both cancer and immune cells possess a significant capacity for adaptation, allowing them to interact and reshape their metabolism to overcome adverse conditions and utilize the metabolic nutrients made differently available. This reprogramming of cancer cell metabolism creates a hostile environment for immune cells, leading to functional defects, primarily impaired effector cell abilities, which provide an advantage to tumor progression (6). Here, we briefly discuss the main metabolic pathways reprogrammed in the TME that facilitate the growth and survival of cancer cells by increasing energy production, antioxidant defense, and the synthesis of macromolecules (8).

### Hypoxia, acidification, and the Warburg effect

The abnormal neovasculature that develops in a growing tumor does not deeply penetrate the tumor tissue (9), resulting in an increased need for oxygen and energy precursors for the synthesis of nucleic acids, lipids, and proteins from the surroundings to the core of the solid tumor (8). Hypoxia-inducible factor 1-alpha (HIF-1α), a transcription factor commonly upregulated in tumors (10), can also be activated by PI3K/Akt/mTOR or MAPK signaling pathways (11) and by oncometabolites such as fumarate and succinate (12). HIF-1α regulates the transcription of various genes, including vascular endothelial growth factor (VEGF), hexokinase 2 (HK2), lactate dehydrogenase (LDH) enzymes, glucose transporters, and carbonic anhydrase IX (CA-IX) for pH regulation (13), among others critical for immune cell function (14). Hypoxia in the TME leads to a metabolic shift in cancer cells from oxidative phosphorylation (OXPHOS) to aerobic glycolysis, known as the Warburg effect (15). This shift increases glucose transport into tumor cells, reducing its availability in the extracellular space while simultaneously increasing lactate and LDH activity (16). Lactate produced by cancer cells is expelled, along with protons, through monocarboxylate transporters (MCTs), inducing TME acidification, further enhanced by CA-IX. CA-IX, with its active site facing the extracellular space, catalyzes CO_2_ hydration, contributing to proton production outside cancer cells. Additionally, the proteoglycan-like domain of CA-IX facilitates the non-catalytic export of protons along with lactate from cancer cells (17). TME acidification provides a growth advantage to cancer cells over immune cells by inhibiting T-cell proliferation, affecting the chemotaxis and migration of neutrophils and dendritic cells (DCs), increasing regulatory T cells (Tregs), and facilitating the infiltration of myeloid-derived suppressor cells (MDSCs) and M2 macrophages. Overall, this supports the immunosuppressive effects on T cells (18) and the TME.

### Role of lipids in tumor and immune cells

Tumor growth is accompanied by a strong demand for lipids and cholesterol, met by increased uptake of exogenous lipids (19), and lipoproteins through promoting lipolysis in adipocytes (8) or by enhancing endogenous synthesis. This lipid metabolism is driven by the overexpression of enzymes in cancer cells for lipid uptake, such as CD36, and transcription factors for lipid oxidation enzymes or lipid synthesis by the tumor cells themselves. Lipids serve not only as an energy source but also play roles in signal transduction and are integral components of cellular structures (20). Specifically, the biosynthesis of cholesterol and phospholipids in tumor cells ensures their survival by maintaining the structural and functional integrity of the cell membrane and facilitating adaptation to the TME, while specific lipids mediate interactions with cells within the TME (21). The role of free fatty acids (fFAs) in immune cell function within the TME is actively investigated, with findings indicating that glycolysis and OXPHOS by long-chain fatty acids can modulate CD8+ Tmem cells to differentiate and promote M2 phenotype polarization (22, 23). CD8+ cells with overexpression of carnitine palmitoyltransferase 1a (Cpt1a), an enzyme catalyzing mitochondrial long-chain fatty acid oxidation (FAO), show a metabolic advantage *in vivo* (23, 24). Recent studies also highlight the necessity of fatty acid (FA) import and Cpt1a-dependent lipid oxidation for maintaining tissue-resident CD9+ cells in peripheral tissues (25), while CD36 is necessary for the increase of CD4+ Tregs in the TME (26).

### Role of amino acids in tumor and immune cells

**Figure 1:**
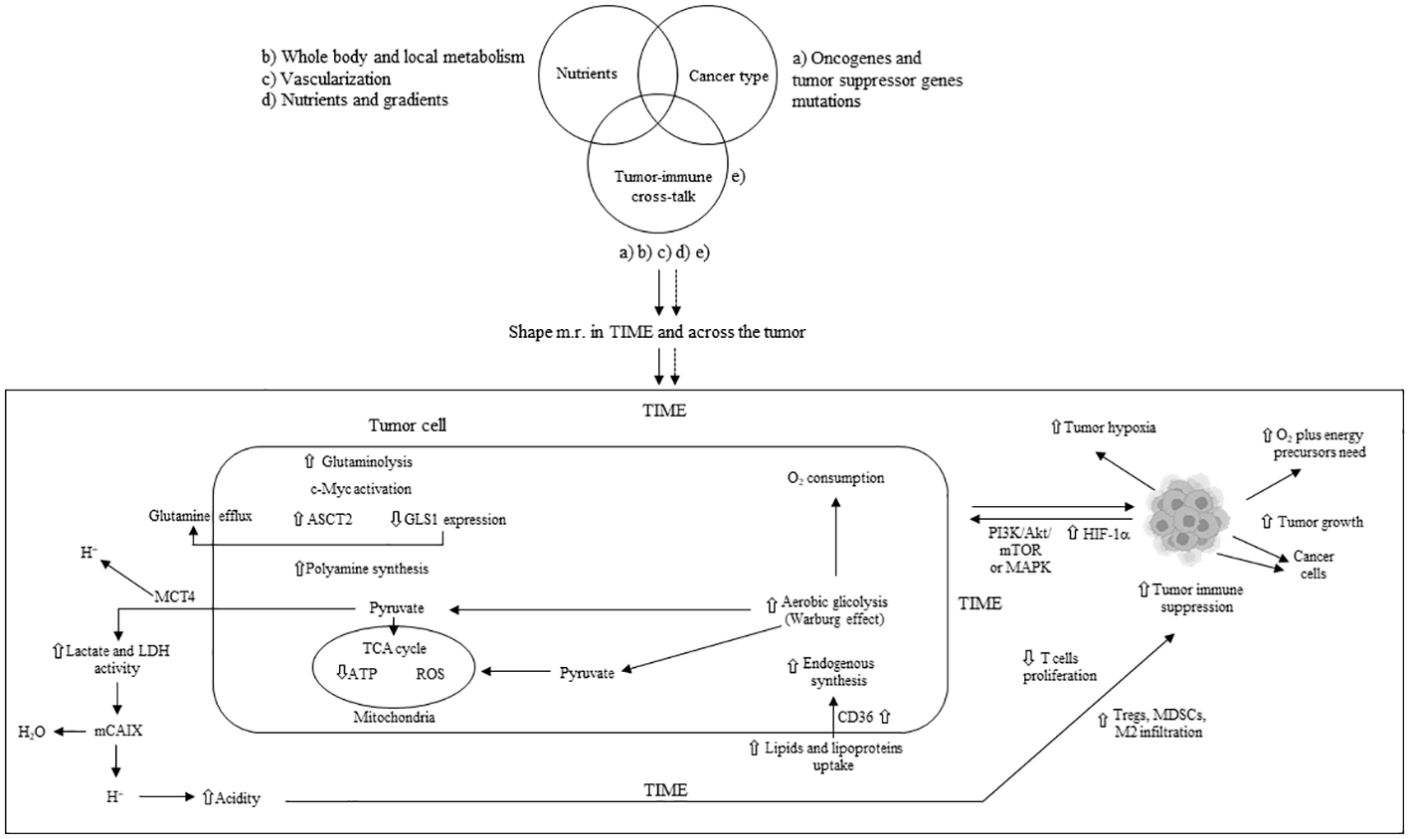
Main mechanisms of metabolic tumor cell reprogramming and tumor immune suppression in TIME. m.r.; metabolism reprogramming; TIME: tumor immune microenvironment; ASTC2 = SLC1A5: solute carrier family 1 member A5; GLS1: glutaminase 1; MCT4: monocarboxylate transporter 4; TCA: tricarboxylic acid; LDH: lactate dehydrogenase; mCAIX: membrane carbonic anhydrase; Tregs: T regulatory cells; MDSCs: myeloid-derived suppressor cells; HIF-1α: hypoxia inducible factor 1 alpha; ATP: adenosine triphosphate; ROS: reactive oxygen species; PI3K: phosphoinositide-3 kinase; Akt: protein kinase B; mTOR: mammalian target of rapamycin; MAPK: mitogen-activated protein kinase; M2: macrophage type 2; ⇧ increase; ⇩ decrease.

In the TME, tumor cells exhibit higher glutamine uptake than infiltrated immune cells (27) leading to a glutamine-deficient TIME. To meet the increased demand for ATP and lipids, tumor proliferating cells enhance the uptake and synthesis of glutamate through glutaminase (GLS), which metabolizes glutamine into glutamate. Tumor cells utilize glutamine to synthesize essential amino acids and produce alpha-ketoglutarate, subsequently generating energy via the tricarboxylic acid (TCA) cycle (28). Receptors for SLC1A5 (ASCT2) and SLC38A5 glutamine transporters are hyper-expressed in some cancers, and their pharmacological inhibition blocks tumor growth (29, 30). The acidic TME activates p53 and increases glucose-6-phosphate dehydrogenase (G6PD) and glutaminase 2 (GLS2) in tumor cells (31). Increased lactate stabilizes HIF-2α, which activates the c-Myc oncogene and leads to the overexpression of the ASCT2 glutamine transporter and glutaminase 1 (GLS1), providing a steady flux of glutamine to the cells (32). Amino acid metabolism homeostasis is also crucial for immune cells, where T-cell activation involves the upregulation of genes encoding amino acid transporters (33). Genetic or pharmacological blockade of GLS inhibits the proliferation and activation of CD4+ and CD8+ T cells, as well as the differentiation of CD4+ Th17 cells (34, 35). GLS overexpression, along with glutamine depletion and elevated glutamate levels, can impair immune cell function (36). The methionine transporter SLC3A2 is hyper-expressed in tumor cells, which compete with CD8+ T cells for methionine import and use (37). Methionine deprivation reduces the methyl donor S-adenosylmethionine (SAM) and then H3K79me2, downregulating STAT5 and impairing the anti-tumor cytotoxicity of CD8+ T cells. Extracellular availability of methionine through one-carbon (1C) metabolism can affect tumor cell growth and T lymphocyte proliferation (38, 39). In T cells, methionine is a crucial amino acid for methylation reactions as the sole origin of the methyl group in SAM (39). Early during T-cell activity, protein synthesis can be affected by alanine levels (40). Tryptophan deprivation counteracts T-cell function through the integrated stress response (41). Upregulation of indoleamine 2,3-dioxygenase (IDO) due to mutations in BIN and KIT oncogenes leads to tryptophan deficiency, inhibiting CD8+ T and NK cells while promoting Tregs and MDSCs, favoring an immunosuppressed TME (42). Cystine uptake and its subsequent intracellular breakdown to cysteine, followed by glutathione synthesis, are critical for activating T cells and for detoxifying reactive oxygen species (ROS) (43). Consequently, a deficiency in cystine hampers T-cell activation and compromises the ROS detoxification process (36). Additionally, the biosynthesis and transport of polyamines are increased in tumor cells, associated with elevated activity of ornithine decarboxylase (ODC), an enzyme essential for carcinogenesis (44, 45). Within the TIME, cancer and immunosuppressive myeloid cells compete with T cells for the uptake and utilization of polyamines. This competition for polyamines can obstruct the proper differentiation of CD4+ T cells and instead facilitate their transformation into immunosuppressive Tregs (14). The primary mechanisms of metabolic reprogramming in tumor cells and the subsequent immune suppression in the TIME are illustrated in **Figure 1**.

### Many mechanisms related to metabolic reprogramming in immune cells facilitate tumor immune escape

#### Metabolic mechanisms of tumor immune escape in T cells

In the TME, pro-inflammatory T effector (Teff) cells, including Th1, Th2, and Th17 phenotypes, primarily rely on increased glycolysis rather than OXPHOS (46, 47). This aerobic glycolysis regulates Teff functions, including IFN-gamma release. In T cells, lactate dehydrogenase A (LDHA) promotes aerobic glycolysis, IFN-gamma production, or Th1 differentiation through epigenetic mechanisms (48). The accumulation of lactate in the TME suppresses essential activities such as the reduction of oxidized nicotinamide adenine dinucleotide (NAD+) to NADH and serine production, crucial for T-cell proliferation (49), and is associated with reduced LDHA expression. Decreased LDHA in T cells inhibits their IFN-gamma-mediated activities and facilitates their conversion to FoxP3+ Tregs. FoxP3 expression in Tregs enhances NAD+ oxidation and shifts their metabolism towards OXPHOS, inhibiting Myc and glycolysis (50), enabling Tregs to thrive in the highly acidic TME (51). Tregs consume more lactate than Teffs, using it to fuel the TCA cycle or gluconeogenesis, thus reducing their glucose requirements in the TME with limited metabolic resources (50, 52). In the TIME, Tregs overexpress MCT-1 for lactate import (52). Moreover, owing to low glucose transporter 1 (Glut1) and high 5-adenosine monophosphate-activated protein kinase (AMPK) expression, Tregs’ immune-regulatory activities depend heavily on FAs or beta-lipid oxidation and OXPHOS, while TME-derived lactate promotes programmed cell death protein 1 (PD-1 or CD279) expression (46, 47). In Tregs, fatty acid binding protein 5 (FABP5) governs OXPHOS and induces immune suppression through IL-10 secretion following type 1 IFN release in a lipid-deficient TME for immune cells (53). FABP5 expression in plasmacytoid dendritic cells (pDCs) induces their tolerogenic function and promotes Treg production (54). Moreover, CD36 upregulation in Tregs, by increasing FA uptake and OXPHOS, favors their survival and proliferation in a highly acidic TIME (26, 55). Concurrently, CD36 expression in CD8+ cytotoxic T cells enhances the uptake of oxidized lipids/low-density lipoproteins (oxLDLs), leading to lipid peroxidation (LPO) (56). and ferroptosis, which are responsible for their death and contribute to immune suppression (57). LPO, p38 mitogen-activated protein kinase (p38MAPK) activity, and impaired mitochondrial biogenesis also promote CD8+ T-cell dysfunction (56, 58). Death/damage-associated molecular patterns (DAMPs) activate Tregs’ Toll-like receptors (TLRs) and FoxP3, which counteract mTORC1 signaling and glucose metabolism, regulating Treg proliferation and immune suppression (59, 60). HIF-1α plays a crucial role in promoting and maintaining glycolysis; its absence in T cells during differentiation redirects them towards a Treg phenotype (47). Tumor-associated Th17 cells with decreased glycolysis are reprogrammed to FoxP3+ Tregs (61). The extracellular accumulation of lactate in the TIME impairs the activation of nuclear factor of activated T-cells (NFAT), IFN gamma production by T and NK cells (62, 63), and the antitumor action of CD4+ and CD8+ cells, also promoting the conversion of CD4+ T cells to Th17 cells (62). Hypoxia, induced by HIF-1α, triggers PDL-1 (CD274) expression in tumor and immune cells, thus promoting immune suppression in the TIME (64). Mitochondrial loss compromises the function of PD-1+ CD8+ T cells in the TIME (65), with a significant correlation observed between the extent of mitochondrial loss and PD-1 expression.

**Figure 2:**
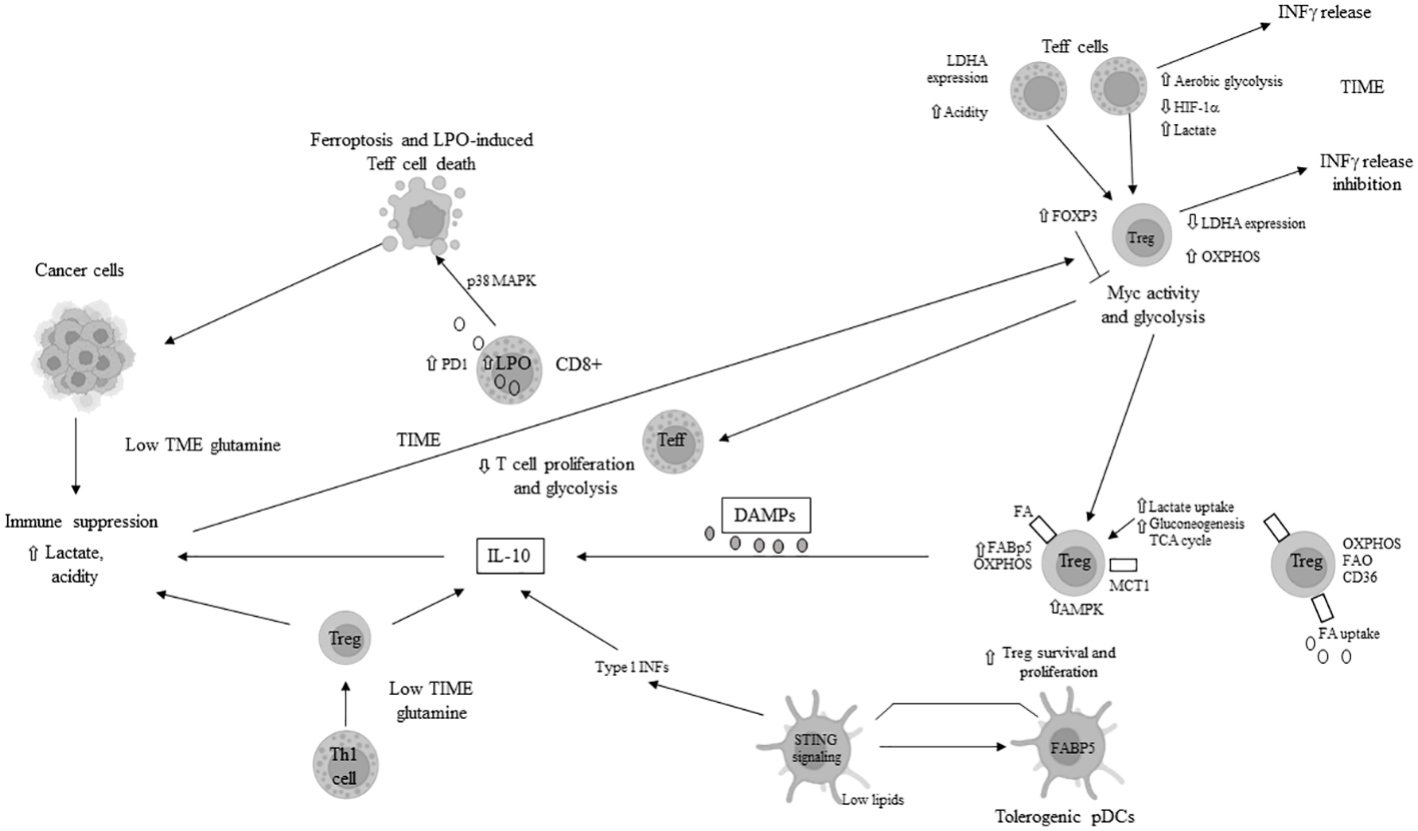
Main mechanisms of tumor immune escape in tumor immune microenvironment (TIME) through metabolic reprogramming in T cells. LDH: lactate dehydrogenase; INFγ: interferon gamma; HIF-1α: hypoxia inducible factor 1 alpha; OXPHOS: oxidative phosphorylation; Treg: T regulatory cell; FABP5: fatty acid binding protein 5; AMPK: AMP-activated protein kinase; TCA: tricarboxylic acid; FA: fatty acid; FAO: fatty acid oxydation; MCT1: monocarboxydate transporter1; PD1: programmed death protein 1; pDCs: plasmacytoid dendritic cells; STING: stimulator of interferon genes; Th: T helper; DAMPs: death/damage-associated molecular proteins; LPO: lipid peroxydase; IL: interleukin; p38MAPK: p38 mitogen-activated protein kinase; ⇧ increase; ⇩ decrease; ⟞ inhibited.

Moreover, the specific nutrient deficiencies in the TIME induce metabolic stress, leading to the failure of Teff cells. Under conditions of hypoxia and mTORC1 signaling inhibition, due to limited glucose availability, there is hypo-expression of antigen-inducing genes, decreased proliferation, and diminished function of CD8+ cell (66, 67), as well as apoptosis of Teff cells triggered by the activation of pro-apoptotic genes/proteins. Additionally, IL-2 signaling-mediated STAT5 activation is impaired in the TME with increased acidity (68), further inhibiting the antitumor function of CD8+ T cells while facilitating the reprogramming of tumor-associated Th17 cells to FoxP3+ Tregs. A glutamine-deficient TIME inhibits the proliferation of infiltrating T cells (69). The lack of glutamine in the TME also reduces cytosolic alpha-ketoglutarate (alpha-KG) in Th1 cells, thereby inducing their differentiation into Tregs (70). PD-1+ CD8+ T cells, owing to enhanced lipolysis of endogenous lipids and FAO, survive longer and promote immune suppression in the TIME. These immune-suppressive CD8+ T cells overexpress markers of lipolysis, including CPT1A, adipose triglyceride lipase (ATGL), and glycerol (71). The primary mechanisms of tumor immune escape through metabolic reprogramming in T cells are illustrated in **Figure 2**.

#### Metabolic mechanisms of tumor immune escape involve macrophages, neutrophils, and myeloid-derived suppressor cells significantly

**Figure 3:**
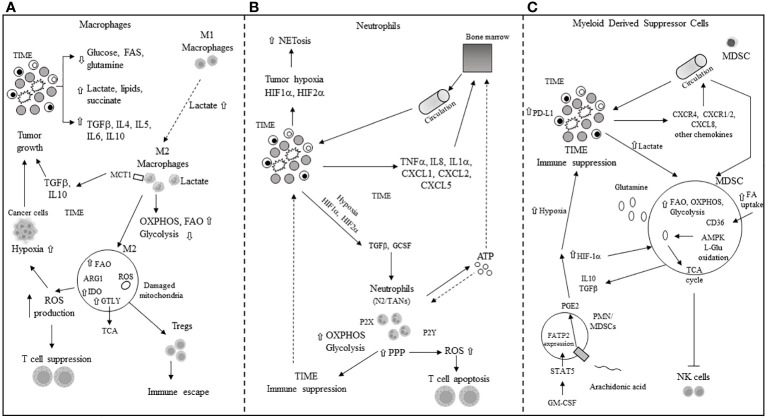
Main mechanisms of immune escape in tumor immune microenvironment (TIME) through metabolic reprogramming in macrophages, neutrophils and myeloid derived suppressor cells. **(A)** M1, M2: macrophage type 1 and type 2 phenotype; TGFβ: tumor growth factor beta; IL: interleukin; FAs: fatty acids; MCT1: monocarboxylic transporter 1; OXPHOS: oxidative phosphorylation; FAO: fatty acid oxidation; ROS: reactuve oxygen species; Tregs: T regulatory cells; IDO: indoleamine 2,3 dioxygenase; ARG: arginase; GTLY: glutaminolysis. **(B)** NETosis: neutrophils extracellular transactivation and release; TNFα: tumor necrosis factor alpha; ATP: adenosin triphosphate; CXCL: chemokine ligand; PPP: pentose phosphate pathway; P2X, P2Y: purinergic receptors; N2: neutrophil type 2; TANs: tumor-associated neutrophils; OXPHOS and ROS: see panel A. **(C)** MDSC: myeloid derived suppressor cell; HIF-1α: hypoxia inducible factor 1 alpha; CXCR: chemokine receptor; AMPK: 5’ adenosine monophosphate activated protein kinase; L-Glu: L-glutamine; CD36: cluster of differentiation 36; TCA: tricarboxylic acid; PGE2: prostaglandin E2; NK: natural killer; GM-CSF: granulocyte-macrophage colony stimulating factor; STAT-5: signal transducer and activator of transcription 5; FATP2: fatty acid transport protein 2; CXCL: see panel B; FAO: see panel A; ⇧ increase; ⇩ decrease.

Pro-inflammatory macrophages (M1) often dominate the immune cell population in the TIME, and a scarcity of glucose, glutamine, and FAs promotes the polarization of M1 into immunosuppressive M2 macrophages or tumor-associated macrophages (TAMs). Elevated levels of TGF-beta, IL-4, IL-5, IL-6, and IL-10 in the TME further induce M2 polarization. The IL-4-driven polarization from M1 to M2 is mediated by the mTORC2-interferon regulatory factor 4 (IRF4) signaling axis, which favors OXPHOS (72) and occurs in the absence of nitric oxide (NO) production due to dysregulated mitochondrial function (73, 74). Interestingly, this IL-4-promoted polarization does not require metabolic reprogramming towards FAO (75). The presence of high lactate, succinate, and other pro-tumor metabolites in the TME further supports the M1-to-M2 polarization through mechanisms such as the yes-1 associated protein (YAP) and NF-kB inhibition via the G protein-coupled receptor 81 (GPR-81)-mediated pathway (76, 77). Overexpression of MCT1 allows macrophages to import lactate, enhancing OXPHOS and FAO, thus favoring the M2 phenotype (78, 79). M2 macrophages secrete immunosuppressive cytokines and chemokines like TGF-beta and IL-10, promoting tumor progression. Tumor cells’ secretion of macrophage colony-stimulating factor (M-CSF) induces fatty acid synthase (FASN) in TAMs (80), which, in turn, promotes the production and secretion of immunosuppressive IL-10. Enhanced lipid availability from tumor cells triggers an endoplasmic reticulum (ER) stress response, leading to increased inositol-requiring enzyme 1 (IRE1) availability and M1 to M2 macrophage polarization and survival in the TIME (81). Elevated levels of IRE1 and STAT3 activation induce the M2 phenotype and immune suppression in the TIME (81–83). M2 macrophage polarization is associated with significant stimulation of OXPHOS in TAMs, mitochondrial damage, and high ROS production (84), further inducing hypoxia and inhibiting the antitumor function of Teff cells. Arginase 1 (Arg1) expression in TAMs depletes L-arginine for T cells and attracts Tregs (85). Concurrently, inducible nitric oxide synthase (iNOS) production in the presence of low arginine can increase ROS and reactive nitrogen species (RNS) levels, thereby facilitating tumor immune escape in the TIME (86, 87). The M2 phenotype is also associated with increased glutaminolysis, supporting the TCA cycle (88). IDO upregulation in M2 macrophages leads to local tryptophan depletion and the production of immunosuppressive kynurenine metabolites (89, 90). Additionally, tumor exosomes in the TME can facilitate the M1-to-M2 conversion by activating the NLRP6/NF-kB signaling pathway (91). While N1 neutrophils maintain antitumor functions, in the TME, pro-tumor N2 neutrophils and the conversion of N1 tumor-associated neutrophils (TANs) to N2 TANs are induced by transforming growth factor-beta (TGF-beta) and granulocyte colony-stimulating factor (G-CSF) (92, 93). In the hypoxic TME, HIF-1α promotes neutrophil survival by enhancing glycolysis (94), while HIF-2α supports the survival of tumor-associated neutrophils (TANs) (95). The pentose phosphate pathway (PPP) in neutrophils increases ROS availability, promoting apoptosis of infiltrating T cells and further immune suppression in the TME (96, 97). The PPP also contributes to neutrophil extracellular trap (NET) formation, or NETosis, by supplying NADPH oxidase with NADPH to generate superoxide, which facilitates cancer spread (98). MDSCs, including monocytic-MDSCs (M-MDSCs) and polymorphonuclear-MDSCs (PMN-MDSCs) (99), inherently promote immune suppression. In cancer patients, PMN-MDSCs appear at very early stages and exhibit enhanced spontaneous migration (100, 101). Various chemokines, including CXCL8, CXCR1/2, and CXCR4, attract MDSCs to the TME (102–104), where they secrete cytokines like IL-10 and TGF-beta, contributing to their immunosuppressive function. Hypoxic conditions in the TME drive MDSCs’ immune-metabolic reprogramming towards FAO and AMPK activation, enhancing their immunosuppressive activity (105, 106). L-glutamine (L-GLn) provided by the TME is metabolized via AMPK, supporting MDSCs’ immunosuppressive activity by fueling the TCA cycle (107). Tumor-associated MDSCs (T-MDSCs) produce their own L-glutamine and, along with upregulated transglutaminase (TGM), enhance their pro-tumor immunosuppressive activity (108, 109). T-MDSCs exhibit increased FAO, OXPHOS, and glycolysis in the TME due to elevated lipid/FA availability through CD36-mediated FA uptake (106). The fatty acid transport protein 2 (FATP2) on PMN-MDSCs, through arachidonic acid (AA) import and prostaglandin E2 (PGE2) production, further enhances their immunosuppressive activity (110, 111). GM-CSF induces FATP2 overexpression in PMN-MDSCs following STAT5 activation. Hypoxia in the TME, alongside high levels of HIF-1α (112, 113), supports the immunosuppressive phenotype of T-MDSCs and induces PD-L1 hyperexpression, inhibiting the cytotoxic and immunological activities of CD8+ and CD4+ T cells (64). Increased lactate in the TME, through the GPR81/mTOR/HIF-1a/STAT3 signaling pathway, supports the survival and proliferation of immunosuppressive MDSCs (114–116), which, in turn, inhibit natural killer cell cytotoxicity (NKCC) (115). The primary mechanisms of immune escape in the TIME through metabolic reprogramming involve macrophages, neutrophils, and MDSCs, as depicted in **Figure 3**.

#### Mechanisms of tumor immune escape in dendritic cells and NK cells

**Figure 4:**
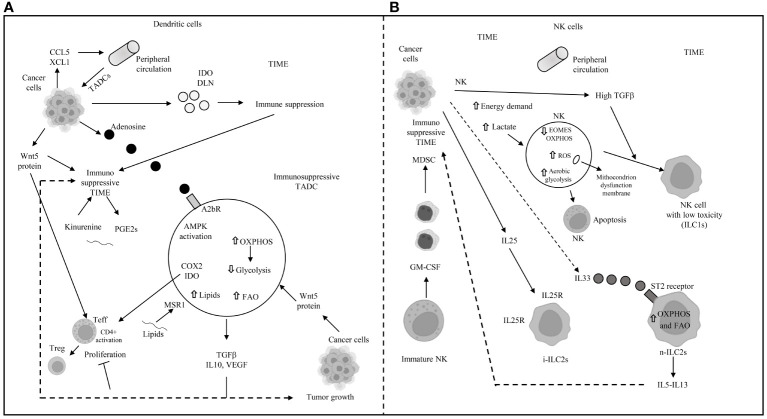
Main mechanisms of immune escape in tumor immune microenvironment (TIME) through metabolic reprogramming in dendritic and natural killer cells. **(A)** DLN: draining lymph-nodes; TADC: tumor-associated dendritic cell; TIME: tumor immune microenvironment; AMPK: 5’ adenosine monophosphate-activated protein kinase; OXPHOS: oxidative phosphorylation; FAO: fatty acid oxidation; COX2: cyclooxygenase2; IDO: indoleamine pyrrole-2,3-dioxygenase; TGFβ: transforming growth factor beta; IL: interleukin; VEGF: vascular endothelial growth factor; MSR1: macrophages scavenger receptor 1; GCN2: general control non depressing 2; A2bR: adenosine A2B receptor; PGE2: prostaglandin E2; CCL5: chemokine (C-C motif) ligand 5; XCL1: chemokine (C motif) ligand 1. **(B)** NK: natural killer; EOMES: eomesodermin; ROS: reactive oxygen species; ILC1s: innate lymphoid cell group 1; i-ILC2s: inflammatory innate lymphoid cells group 2; n-ILC2s: natural innate lymphoid cells group 2; GM-CSF: granulocyte-macrophage colony stimulating factor; MDSC: myeloid derived suppressor cell; IL: interleukin; IL25R: interleukin 25 receptor; OXPHOS and FAO: see panel A; ⇧ increase; ⇩ decrease; —I inhibited.

In the competitive TIME, AMPK overexpression in tumor-associated dendritic cells (TADCs) enhances FAO and OXPHOS, transforming them into tolerogenic DCs (117, 118). Their essential antigen-presenting function is compromised by an abnormal increase in lipids, imported from the extracellular space through macrophage scavenger receptor 1 (MSR1) (119, 120). The binding of exogenous adenosine monophosphate (AMP) to the adenosine A2b receptor on TADCs elevates their pro-tumor functions, creating an immunosuppressive environment through the secretion of VEGF, TGF-beta, IL-10, and the expression of cyclooxygenase-2 (COX-2) and IDO (121, 122). IDO mediates the metabolic production of kynurenine (123) from tryptophan, while the decrease in tryptophan inhibits T-cell proliferation and encourages the transition of naïve CD4+ T cells to FoxP3+ Tregs (41–126). Kynurenine and other metabolites activate the aryl hydrocarbon receptor (AhR) on T cells, inducing Treg differentiation and promoting an immune-suppressive phenotype in macrophages and DCs (126–129). Wnt5 protein, secreted by tumor cells, activates the peroxisome proliferator-activated receptor (PPAR)-gamma through beta-catenin, inhibiting the immune-metabolic shift to glycolysis and activating FAO by upregulating CPT1A in TADCs. Beta-catenin also enhances vitamin A metabolism in TADCs, with retinoic acid (RA) production further inducing Tregs and supporting an immune-suppressive TIME (130). Innate lymphoid cells (ILCs) include group 1 ILCs, which encompass NK cells. In NK cells, an increased energy demand prompts immune-metabolic reprogramming towards aerobic glycolysis to support their function, though mTORC1 activity also leads to an increase in OXPHOS (131, 132). However, the high demand for glucose is not met in the TIME, where elevated TGF-beta impairs mitochondrial metabolism and OXPHOS, inhibiting NK cell function (133). TGF-beta, through mTOR inhibition, hampers NK cell proliferation and maturation, which are promoted by IL-15 (134), while blocking TGF-beta restores NK cell anti-tumor function (133, 134). High lactate levels in the TME promote mitochondrial dysfunction and increased ROS, inhibiting NK cells’ OXPHOS and leading to their apoptosis due to energy deficiency (135). In the presence of TGF-beta, secreted by tumor and suppressive immune cells, NK cells in the TIME can avoid apoptosis and transition to less cytotoxic ILC1s, which survive on lower energy requirements. GM-CSF in the TIME also converts immature NK cells to MDSCs, promoting cancer metastasis and spread (136). TGF-beta in the TME is responsible for the hypo-expression of eomesodermin (EOMES) (137), crucial for NK cell development and cytotoxicity (138, 139), and reprograms anti-tumor ILC3s to pro-tumor regulatory ILC3s (ILCregs) that release IL-10 (140). IL-25, part of the IL-17 cytokine subfamily, converts inflammatory ILC2s (iILC2s) to natural ILC2s (nILC2s), which overexpress IL-25R+ and are responsible for increased secretion of IL-5 and IL-13, contributing to an immunosuppressive TIME (141). In CRC, inhibition of IL-25R decreases tumor growth and triggers an immune response against the tumor in mice (142). IL-33 exerts pro-tumor activity, including PPAR-gamma-mediated delivery of IL-4, IL-13, and IL-15 from ILC2s (143). Elevated levels of IL-33 in the TIME, through binding to its receptor ST2, lead to the transient accumulation of imported FAs in lipid droplets, inducing ILC2’s pro-tumor activity (144). The primary mechanisms of immune escape in the TIME through metabolic reprogramming in dendritic and natural killer (NK) cells are depicted in **Figure 4**.

## Metabolic reprogramming in CRC

In CRC, the TME facilitates metabolic intercommunication among various cells, leading to abnormal glycolysis, glutaminolysis, and lipid synthesis (145), largely in line with previous descriptions and partly regulated by genetic and epigenetic phenotypes. Additionally, the intestinal microbiota plays a crucial role in this intercellular metabolic cross-talk. This summary encapsulates the primary findings related to the reconfiguration of metabolic pathways in CRC.

### Glycolysis and glutaminolysis

Aerobic glycolysis, also known as the Warburg effect, has been observed in CRC (146). The enzyme pyruvate kinase (PK) is crucial for glycolysis (147), with the altered form PKM2 (PK muscle isozyme 2) (148, 149), increased lactate production, and the upregulation of glucose transporter 1 (GLUT1), Hk2, and other glycolytic enzymes being reported in CRC (150–152). Additionally, mutations in several enzymes related to the TCA cycle are linked to poor outcomes in CRC. Glutamine, transported into the cell cytosol and facilitated by SLC1A5 (also known as ASCT2, an alanine, serine, cysteine transporter 2) (153, 154), is overexpressed in CRC (155), promoting cell survival and proliferation (156). Serine, a non-essential amino acid involved in 1C metabolism, is crucial for the proliferation of CRC cells (157–159). The enzyme IDO, which converts tryptophan to kynurenine, is upregulated in CRC and correlates with tumor growth and patient outcomes (160). 1C metabolism, involving the folate and methionine cycles, generates 1C groups necessary for synthesizing vital precursors, such as purines and pyrimidines, and for methylation processes (161). Folate, a form of vitamin B9 obtained from the diet, transfers 1C groups from inside to outside the cells (157). There is an upregulation of folate-dependent 1C metabolic enzymes, including folate receptor-1 (FOLR1), dihydrofolate reductase (DHFR), and serine hydroxymethyltransferase 1 (SHMT1) in CRC cells, unlike in non-transformed cells (162, 163). Increased SHMT2 expression, which becomes stabilized through NAD-dependent deacetylase sirtuin-3 (SIRT3)-mediated deacetylation (164), is associated with a worse prognosis in CRC patients (164). Overexpression of methylenetetrahydrofolate dehydrogenase 1-like (MTHFD1L) has been found in primary CRC patients, and its suppression inhibits colon cancer growth and spread (165). The upregulation of SHMT2, MTHFD2 enzymes, and mitochondrial 10-formyltetrahydrofolate dehydrogenase (ALDH1L2) distinguishes CRC tissues from normal controls (166). Oxamate, 2-DG, and lonidamide, as glycolytic inhibitors, along with 6-diazo-5-oxo-L-norleucine (DON) and CB839 as glutaminolysis inhibitors have been extensively researched for their potential in CRC treatment (**Table 1**). The role of protein N-homocysteinylation (N-Hcy) in CRC has also been documented (191). Studies involving high-risk CRC patients and animals have shown that a high-fat diet can elevate homocysteine (Hcy) levels (192–194). Progressing CRC patients have been found to have higher N-Hcy plasma levels compared to healthy controls (194). Additionally, there is an observed upregulation of N-Hcy-protein and methionyl-tRNA synthetase (MARS) in CRC tissues versus normal tissues (194), along with changes in protein structure and function due to irregular N-Hcy, particularly affecting DNA damage repair proteins such as ataxia telangiectasia and Rad3-related protein (ATR) (194). These alterations lead to increased microsatellite instability and promote diffusion in cancer cells (194). ATR K-Hcy is associated with increased DNA damage and enhances CRC cell survival and growth (194). Inhibiting the production of Hcy-thiolactone (HTL), which is facilitated by MARS, thereby reducing K-Hcy changes, has been shown to decrease DNA damage and CRC cell proliferation. This suggests that MARS inhibitors could be beneficial in CRC therapy (194).

**Table 1:** Main components/agents targeting glucose, glutamine and lipid metabolism for CRC treatment.

Metabolic target	Metabolic agent/compound	IT combined	Immune cells	Cancer cells	Mechanism	Ref
Glucose metabolism	PEP, GAPDH	No	Metabolic checkpoint regulation	Indirect action	ACT through T-cell reprogramming	(8)
	Oxamate	No	No	Tumor growth inhibition	LDHA inhibitor; synergistic effect when combined with mTOR inhibitor rapamycin	(167)
	2-DG	No	Tregs depletion, Th2 and Th17 shift to Th1, polarization to M1	Increased local tumor control	Tumor cells sensitized to radiation and CT drugs following ATP and NADPH decrease due to HK inhibition	(8, 168)
	2-DG and 6-AN	No	As above	As above	Increased radiosensitization	(8)
	2-DG, Metformin, Caulerpin	No	FOXO1 decrease; memory T cells and CD8+ increase	Indirect action	AMPK activity increase and negative mTOR regulation (2-DG and Caulerpin); c-myc downregulation (Metformin)	(8, 169–172)
	Quercetin, 3-BP, Lonidamide	No	^a^No	Tumor growth inhibition	MCT1 inhibition	(173–175)
	Indisulam (E7070)	No	No	Tumor growth inhibition	Multiple CAs inhibitor; synergize with capecitabine and irinotecan	(176, 177)
	Bortezomib, Irinotecan, EZN-2208	No	No	Tumor growth inhibition	HIF1 inhibitors	(177)
	Aspirin, Apatinib, Trametinib	With ICB	Teff cells activity increase	Tumor growth inhibition	The combination of these glycolytic inhibitors with ICIs can be helpful in reversing the resistance to single agent ICI	(178–180)
Glutamine metabolism	6-diazo-5-oxo L-norleucine (DOS)	No	No	Tumor growth inhibition	Inhibitor of glutaminolysis and induction of cellular ROS	(181)
	CB839	No	No	Tumor growth inhibition	Inhibitor of glutaminolysis; combination with cetuximab	(181)
Lipid metabolism	Cerulenin	No	No	Tumor growth inhibition	FASN inhibitor; suppression of CRC cell proliferation, apoptosis induction and inhibition of metastasis	(182, 183)
	Luteolin (3,4,5,7-tetrahydroxyflavone)	No	No	Tumor growth inhibition	FASN inhibitor; modulation of IGF-1 and Wnt-beta-catenin oncogenic pathways	(184)
	EGCG (epigallocatechin-3-gallate)	No	No	Tumor growth inhibition	FASN inhibitor; CRC cell proliferation and diffusion inhibition through STAT3 downregulation	(181)
	TOFA	No	No	Tumor growth inhibition	ACC inhibitor; induction of CRC cell apoptosis	(185)
	^b^CD36 inhibitor	Anti PD-1 treatment	Tregs dysfunction	Tumor growth inhibition	Increased efficacy of anti-PD-1 therapy following CD36 inhibition in intratumor Tregs	(26, 57)
	^b^cPLA2-alpha inhibitor	Adaptive T cell transfer therapy	Teffs	Indirect action	Prevent the dysfunction and senescence of Teff cells	(186)
	Bezafibrate	Anti PD-1 treatment	Teffs	Indirect action	FAO increase to prevent cell death due to FAO inhibition of anti-PD-1 therapy	(187–189)
	AZD1208	Anti PD-L1	MDSCs	Indirect action	PIM1 inhibitor, which inhibits FA uptake and FAO in MDSCs to increase MDSCs and recruit Teff cells	(190)

CRC, colorectal cancer; IT, immunotherapy; PEP, phosphoenolpyruvic acid; GAPDH, glyceraldehyde-3-phosphate dehydrogenase; ACT, adoptive cell therapy; 2-DG, 2-deoxy-D-glucose; LDHA, lactate dehydrogenase A; Th, T helper cell; M1, macrophage phenotype 1; CT, chemotherapy; ATP, adenosine triphosphate; HK, hexokinase; 6-AN, 6-aminonicotinamide; FOXO1, forkhead box protein 01; AMPK, 5-adenosine monophosphate-activated protein kinase; 3-BP, 3-bromopyruvate; MCT, monocarboxylate transporter; CAs, carbonic anhydrases; HIF, hypoxia-inducible factor; ICB, immune checkpoint blockade; ROS, reactive oxygen species; ICIs, immune checkpoint inhibitors; FASN, fatty acid synthase; IGF1, insulin growth factor 1; STAT-3, signal transducer and activator of transcription 3; ACC, acetyl-CoA carboxylase; CD36, cluster of differentiation 36 also known fatty acid translocase (FAT); PD-1, programmed death-1; FAO, fatty acid oxidation; MDSCs, myeloid-derived suppressor cells; PIM1, proto-oncogene serine/threonine-protein kinase; FA, fatty acid. ^a^Recovery of ICB resistance in murine models; ^b^not available.

### Lipid synthesis

High levels of lipids and increased lipogenesis have been identified in CRC cases with poorer prognoses (19, 195, 196), with 24 distinct lipids among 36 metabolites showing differential expression in adenocarcinomas compared to non-adenocarcinomas (196). Alterations in FA metabolic pathways have been linked to tumor growth and worse outcomes in CRC (197, 198). The activation of CD36 has been shown to inhibit CRC growth and induce apoptosis (199). Notably, CD36 overexpression is more common in CRC metastases than in primary CRC, indicating a greater dependency on FA uptake in metastatic cells (200–202). Overexpression of FABP5 enhances CRC growth and spread through a PPAR β/δ-independent signaling pathway (203). Within cells, long-chain acyl-CoA synthetases (ACSLs) and very long-chain acetyl-CoA synthetases (ACSVL) activate FAs by coupling them with CoA, while the concurrent upregulation of ACSL1, ACSL4, and SCD1 promotes epithelial-to-mesenchymal transition (EMT) in CRC (204).

Carnitine palmitoyltransferase 1 (CPT1), by interacting with carnitine, converts acyl-CoA into acyl carnitine, which is then transformed back into acyl-CoA by carnitine palmitoyltransferase 2 (CPT2) inside the mitochondria (205). In FAO, acetyl-CoA is the final product of acyl-CoA and then enters the TCA cycle (205), with CPT1A being more expressed in metastatic than in primary CRC (201). Citrate, derived from the TCA cycle, is converted into acetyl-CoA and oxaloacetate by ATP-citrate lyase (ACLY). In the cytoplasm, acetyl-CoA is transformed into malonyl-CoA, which are then condensed by fatty acid synthase (FASN). Wen et al. found that ACLY induces CRC growth inhibition by promoting β-catenin stabilization and its nuclear transport and transcriptional activity (206), although ACLY also induced CRC metastasis *in vivo* (206). Upregulation of FASN enhances CRC growth and spread through the AMPK/mTOR signaling pathway and is associated with secondary lymph node involvement, tumor stage (TNM), and poorer CRC outcomes (207).

**Figure 5:**
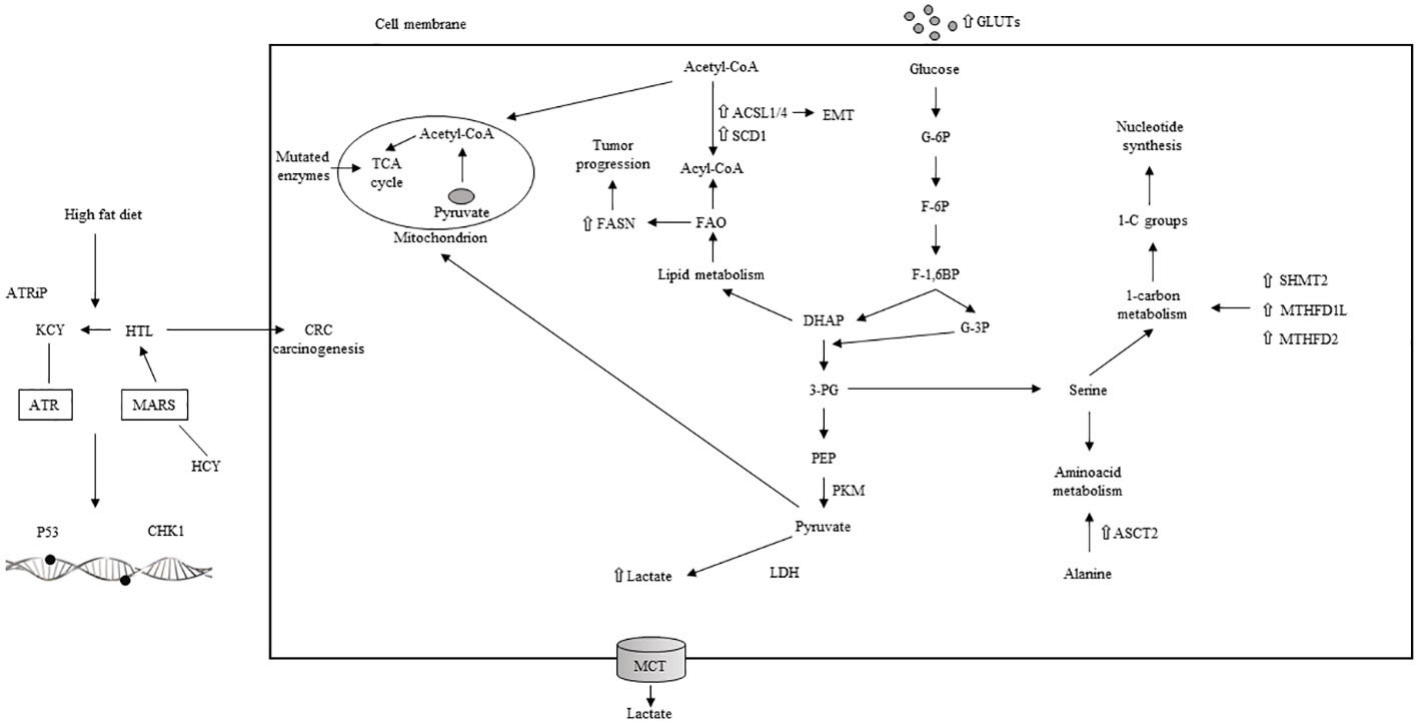
High fat diet, aerobic glycolisis and main characteristics of metabolic reprogramming in CRC. TCA: tricarboxilic acid; ACSL: acyl-CoA synthetase; SCD: stearoyl-CoA desaturase; EMT: epithelial to mesenchymal transition; FAO: fatty acid oxydation; DHAP: dihydroxyacetone phosphate; G-6P: glucose 6 phosphate; F-6P: fructose 6 phosphate; F-1,6BP: fructose 1,6 biphophate; G-3P: glucose 3 phosphate: 3-PG: 3 phosphogliceric acid; PEP: phosphoenolpyruvate; PKM: pyruvate kinase muscle isoenzyme; LDH: lactate dehydrogenase; MCT: monocarboxylate transporter; 1-C: one carbon; SHMT: serine hydroxymethyltransferase; MTHFD1L: methylene tetrahydrofolate dehydrogenase 1-like; ASCT: alanine, serine, cysteine transporter; ATRiP: ATR interacting protein; KCY: colonic lysine homocysteinylation; ATR: ataxia-teleangectasia and RAD 3-related protein; HTL: homocysteine thiolactone: MARS: methionyl-tRNA synthetase; CHK1: cheek point kinase 1; HCY: homocysteilation; ⇧ increase; ⇩ decrease.

The metabolic axis of stearoyl-CoA desaturase (SCD)/ACSLs induces EMT in CRC cells and, in stage II CRC patients, is associated with worse prognosis (204). Inhibiting SCD along with ACSLs reduced the viability of CRC cells without affecting normal cells (204). Elevated serum cholesterol has been linked to a higher rate of CRC (208, 209). Stage III–IV CRC patients overexpressing 3-hydroxy-3-methylglutaryl-CoA reductase (HMGCR) (209) showed better clinical outcomes. Conversely, the upregulation of ABCA1, an ATP-binding cassette transporter involved in cholesterol and phospholipid homeostasis, was associated with worse CRC outcomes by promoting tumor proliferation and caveolin-1-dependent spread (210). A high-fat diet and the main features of metabolic reprogramming in CRC are illustrated in **Figure 5**. Lipid metabolism inhibitors such as FASN, ACC, CD36, and cPLA2-alpha have been extensively investigated for CRC treatment.

## The genetic and epigenetic phenotype regulate metabolic reprogramming

Mutations in the adenomatous polyposis coli (APC) gene, which are present in over 80% of sporadic CRC cases, lead to increased glycolysis (211, 212). These APC mutations result in the activation of β-catenin/T-cell factor (TCF) transcription, subsequently increasing the expression of cMYC, PKM2, pyruvate dehydrogenase kinase 1 (PDK1), and MCT1 genes (211–214). PDK1, a glycolytic enzyme, inhibits the conversion of pyruvate into acetyl-CoA, thereby reducing OXPHOS in mitochondria and maintaining aerobic glycolysis in cancer cells (215). The APC/β-catenin axis also regulates HIF-1α and MYC transcription factors (214), with aberrant activation of β-catenin via HIF-1α promoting metabolic reprogramming of glucose in CRC (216, 217). Genetic aberrations in the TP53 gene, including inactivating mutations or deletions, occur in 40%–50% of sporadic and 80% of advanced CRC cases (218, 219). p53 modulates glucose metabolism by inhibiting transcription and transportation of GLUT1, GLUT3, and GLUT4, reducing glucose import (220). It also regulates the expression of glycolytic enzymes Hk2 and fructose 2,6-biphosphate (F2,6BP), thereby decreasing glycolysis (221). Additionally, p53 reduces intercellular lactate translocation by downregulating MCT1 (222) and interacts with HIF-1α and MYC (223), promoting the degradation of HIF-1α through parkin overexpression (224, 225) and suppressing c-myc transcription (226). In cancer cells with p53 mutations, the lack of inhibition on HIF-1α and MYC leads to their accumulation. Activating mutations in RAS and hyper-expression of EGFR occur in approximately 50% and 80% of CRC cases, respectively, leading to the continuous activation of the PI3K/Akt/mTORC1 pathway (227, 228), which alters cancer metabolism (229). AKT and mTORC1 enhance glycolysis by increasing glucose import and phosphorylating glycolytic enzymes. PI3K/Akt pathway also promotes MYC transcription and increases HIF-1α and MYC proteins by inhibiting their degradation and facilitating their translation (229, 230). Methylation, both epigenetic and genetic, plays a key role in regulating glycolytic metabolism (231). DNA methylation activates aberrant glycolytic metabolism in cancer (232). For example, hypermethylation of the LDHB promoter increases the LDHA/LDHB ratio, enhancing lactate formation in cancer cells (232). Hypomethylation of the HK2 promoter leads to HK2 overexpression and tumor progression (233). DNA methylation also upregulates HIF-1α, enhancing HIF pathway signaling in cancer (232). In CRC, N6-methyladenosine (m6A) modification of Hk2 and SLC2A1 mRNA by METTL3 stabilizes them, promoting the glycolytic pathway (234). Methylation of glycolytic enzymes LDHA and PKM2 by methyltransferases regulates their function (235, 236), underscoring the significant role of methylation in CRC glycolytic metabolism.

**Figure 6:**
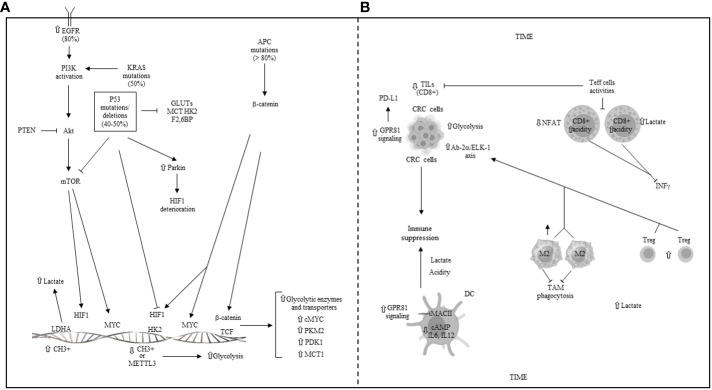
**(A)** Genetic and epigenetic regulation of glycolysis in CRC. EGFR: epidermal growth factor receptor; APC: adenomatous polyposis coli gene; PI3K: phosphoinositide-3-kinase; Akt: protein kinase B; mTOR: mammalian target of rapamycin; PTEN: phosphatase and tensin homolog; KRAS: kirsten and sarcoma virus gene; P53: tumor protein 53; GLUTs: glucose transporters; MCT: monocarboxylate transporter; HK2: hexokinase 2; F2,6BP: fructose 2,6 biphosphate; HIF1: hypoxia inducible factor 1; PKM: pyruvate kinase muscle isoenzyme; PDK1: piruvate dehydrogenase kinase 1; TCF: T cell factor; METTL3: methyl adenosine (m6A) transferase; LDHA/B: lactate dehydrogenase A/B. **(B)** Metabolic reprogramming and some main mechanisms of immune escape in the colorectal tumor immune microenvironment (TIME). TILs: tumor infiltrating lymphocytes; CRC: colorectal cancer; GPR81: G-protein coupled receptor 81; Ap2α: activating protein 2 alpha; Elk-1: ETS-like 1 protein; DC: dendritic cell; NFAT: nuclear factor of activated T cells; M2: macrophage phenotype 2; Treg: T regulatory cells; TAM: tumor-associated macrophage; ⇧ increase; ⇩ decrease; ⟞ inhibited.

Genetic and epigenetic regulation of glycolysis in CRC, illustrated in **Figure 6A**, is often associated with various signaling pathways, including Wnt-beta-catenin, EGFR/RAS/RAF/MAPK, PI3K, VEGF, and p53, which contribute to CRC initiation and progression. Targeted therapies against these pathways, such as cetuximab (anti-EGFR), encorafenib (BRAF inhibitor), and bevacizumab (anti-VEGF), have been successfully administered for treating metastatic CRC patients.

## The metabolic immune escape in CRC

In CRC, the competition for nutrients within the TME triggers a reprogramming of key metabolic pathways, which, in turn, promotes immune escape. The metabolites produced by the intestinal microbiota offer an additional explanation for immune tolerance.

### Reprogramming of key metabolic pathways

Within the TME, both tumor cells and other cells exhibit increased lactate production due to the Warburg effect, leading to a highly acidic environment. This acidosis, similar to that observed in other cancers, impedes the activity of anti-tumor immune cells in CRC (237). Specifically, lactate diminishes the functionality of CD8+ T cells (63), with studies showing that CD8+ T cells exposed to acidic conditions undergo intracellular acidification via lactate uptake (63), significantly reducing the activity of activated T cells (NFAT), a crucial transcription factor, and the production of IFN-γ, a critical cytokine for immune response (63). In CRC with deficient mismatch repair (dMMR)/microsatellite instability-high (MSI-H), proteomic analyses have revealed an inverse relationship between glycolytic enzyme levels and the presence of infiltrating CD8+ T cells (238). High glycolysis levels in MSI-H CRC samples correlate with fewer infiltrating CD8+ T cells, suggesting that glycolysis may influence the efficacy of immune checkpoint inhibitor (ICI) therapy and could predict the prognosis of CRC patients undergoing immunotherapy (238). The scarcity of nutrients within the TME fosters an immune-suppressive phenotype, characterized by Tregs and M2-like macrophages (239). Furthermore, lactate produced by CRC cells inhibits the phagocytosis of TAMs by activating the Ap-2α/ETS-like-1 protein (Elk-1) axis, which increases levels of signal-regulatory protein alpha (SIRPα), dampening TAMs’ anti-tumor activity (240). Conversely, reducing lactate levels can enhance the effectiveness of immune therapy in CRC by diminishing the presence of tumor-infiltrating Tregs and MDSCs (241). Lactate also serves as an agonist for the GPR81, which, when activated in cancer cells, leads to PD-L1 overexpression and facilitates tumor immune escape (242). In DCs, lactate activation of GPR81 inhibits MHC class II presentation on the cell surface and reduces the production of cAMP, IL-6, and IL-12, further contributing to immune evasion (243). An increase in GPR81 signaling not only diminishes the pro-tumor activities of TAMs but also increases the immune-suppressive capabilities of MDSC (77). Recent studies have further highlighted the critical role of lactylation in modulating immune cell activity within the hypoxic and acidic TME (244). Lactate-induced histone lactylation can elevate METTL3 expression in tumor-infiltrating myeloid cells (TIMs) (245), activating JAK/STAT signaling pathways, thereby fostering an immune-suppressive TME and promoting tumor growth and spread (245). Decreased density of mature DCs has been observed in both human colon cancers and experimental rat colon cancer models, with an even lower DC density in metastases compared to primary colorectal tumors. Additionally, various factors secreted by tumors and on a systemic level contribute to the functional defects of tumor-infiltrating DCs in CRC. Factors such as CCL2, CXCL1, CXCL5, and VEGF are implicated in inhibiting IL-12p70 secretion by DCs, while COX2 impedes DC differentiation and maturation through the mediation of the downstream signal molecule PGE2 and its receptors EP2/EP4 (246). Apoptosis is a mechanism for DC elimination in the TME, with MUC2 mucins increasing the rate of apoptosis in cultures of DCs derived from human monocytes. It has also been reported that the number of CD205-positive intra-tumor DCs decreases in patients with CRC. Furthermore, upregulation of HMGB1, a multifunctional cytokine secreted by cancer cells, is associated with lymph node metastasis, suggesting that nodal DCs are suppressed by HMGB1 produced by colon cancer cells (247). Regarding NK cells, various mechanisms have been identified for escaping NK cell-mediated tumor surveillance. Fucosylation plays significant roles in carcinogenesis and is among the most critical oligosaccharide modifications in cancer (248). Research has shown that mutations in the GDP-mannose-4,6-dehydratase (GMDS) gene, essential for fucosylation, result in resistance to TRAIL-induced apoptosis and subsequent evasion from NK cell-mediated surveillance in human colon cancer. Additionally, a more recent study (249) focused on the long non-coding RNA (lncRNA) ELFN1-AS1, which is aberrantly expressed in multiple tumors and considered an oncogene in cancer development. This study revealed that ELFN1-AS1 increases the ability of CRC cells to evade NK cell surveillance both *in vitro* and *in vivo* by attenuating NK cell activity. It achieves this by downregulating NKG2D and GZMB through the GDF15/JNK pathway. Metabolic reprogramming and several primary mechanisms of immune escape within the colorectal TIME are depicted in **Figure 6B**.

### The dual role of the microbiota

Intestinal cancer cells and other components of the TME interact with the microbiota, which plays a significant role in reshaping the TME (250). Metabolites from the microbiota, such as short-chain fatty acids (SCFAs) and lipopolysaccharide, influence the TME. CRC is characterized by high levels of *Fusobacterium nucleatum* and low levels of *Holdemanella biformis*, which promote anti-tumor activity by enhancing SCFA formation (251, 252). Conversely, dysbiosis is associated with decreased levels of SCFAs and polyamines. SCFAs, including butyrate, acetate, and propionate, are essential for maintaining intestinal homeostasis (251, 253). Butyrate and acetate, in particular, are known to increase the activity of CD8+ effector T cells (Teff) through direct effects (254, 255) and epigenetic mechanisms (256). Polyamines, however, have been shown to create an immunosuppressive TME by reducing IFN-gamma and TNF levels, though contrasting effects of polyamines have also been reported (257). Among polyamines, spermine favors the M2 polarization of TAMs, while spermidine promotes an M1 phenotype (258).

An experimental study on CRC (259) explored how SCFA treatment affects the ability of CRC cells to activate CD8+ T cells. SCFAs, as microbial metabolites, possess immune-regulatory properties within CRC cells that are not fully understood. SCFA-treated CRC cells were found to activate CD8+ T cells more effectively than untreated cells. Additionally, SCFAs activated CD8+ T cells more in CRCs with microsatellite instability (MSI) compared to chromosomally unstable (CIN) CRCs without DNA repair deficiency. This suggests that SCFAs, depending on the genotype of CRC cells, may induce DNA damage that promotes the upregulation of chemokines, MHC class I, and genes involved in antigen processing or presentation. An increase in response is facilitated by a direct feedback loop between SCFA-treated CRC cells and activated CD8+ T cells in the TME. In CRCs, the suppression of histone deacetylation by SCFAs initiates a process that leads to genetic instability and the overexpression of genes likely involved in SCFA signaling and chromatin regulation. This effect is observed in MSI CRC samples, regardless of the quantity of SCFA-producing bacteria in the bowel. These findings indicate that the heightened response of MSI CRCs to microbially delivered SCFAs significantly enhances CD8+ T-cell activity, thus identifying a potential therapeutic target to boost anti-tumor immunity in CIN CRCs (259).

## The immunotherapy with ICIs

Recently, immunotherapy using ICIs has emerged as a promising therapeutic option for selected CRC patients. Nivolumab and pembrolizumab, ICIs that have shown success in treating patients with MSI-H (microsatellite instability-high) colorectal tumors, are notable examples (260). However, the immunosuppressive TME in most CRC patients likely impedes the effectiveness of immunotherapy. As mentioned earlier, effector T cells require ample nutrients to sustain their antitumor activity, and glucose deprivation compromises their presence and functionality (261). Furthermore, lactic acid (LA), a metabolic by-product prevalent in the competitive TME, significantly inhibits T cell-mediated lysis of cancer cells (261). Recent studies have shown that an acidic TME enhances the expression of PD-1 and other suppressive molecules in Tregs, while dampening PD-1 expression in effector T cells due to Tregs’ preferential utilization of lactate (262). This suggests that PD-1 blockade might inadvertently bolster PD-1+ Treg cell function, thereby facilitating resistance to immunotherapy (262). An inverse relationship between tumor glycolysis and tumor-infiltrating CD8+ T cells has been observed (238), with high serum LDH levels predicting poor responses to pembrolizumab (263). Research in a murine model of mismatch-repair-proficient (pMMR) CRC confirmed the inverse association between the therapeutic efficacy of PD-1 inhibitors and serum LDH levels (263, 264), with patients experiencing enhanced effectiveness of PD-1 blockade when combined with LDHA inhibition (178). Despite the metabolic overlap between cancer and immune cells in the highly competitive TME, subtle differences exist. Drugs targeting glycolysis can inhibit both pro-tumor and anti-tumor immune cells (265). Specifically, treatment with 2-DG has been shown to significantly suppress key activities in CD4 and CD8 T cells, such as cell proliferation and lactate production (266) and further inhibit the production of IFN-γ, TNF, IL-10, and IL-4 in CD4 T cells (266). Additionally, effector T cells deficient in glucose transporter 1 (Glut1) were unable to augment and promote an inflammatory response (267), unlike Treg cells, which retained their functionality in a Glut1-independent manner (267). This underscores the importance of precisely defining the subtle differences among subpopulations of the same cell type, particularly immune cells, to optimally exploit their vulnerability to metabolic inhibition.

## Clinical applications of the metabolic reprogramming and ICIs

### Targeting ICIs and/or glucose metabolism

Targeting ICIs and/or glucose metabolism involves identifying specific metabolites, metabolic enzymes/pathways, and genes that are differentially expressed or regulated in tumor and immune cells. The goal is often to enhance the antitumor activity of effector T cells (Teff) while diminishing the immunosuppressive functions of Tregs, without adversely affecting their functionality. Focusing on glucose metabolism, the glycolytic metabolite phosphoenolpyruvic acid (PEP) can influence the activity of tumor-infiltrating lymphocytes (TILs), and enzymes such as glyceraldehyde-3-phosphate dehydrogenase (GAPDH) serve as metabolic checkpoint regulators. Reprogramming T-cell metabolism by altering the levels of these metabolites and enzymes can act as an adoptive cell therapy (ACT) and supplement immunotherapy (8). Inhibiting key enzymes is another primary therapeutic approach. For instance, oxamate, an inhibitor of lactate dehydrogenase A (LDHA), has been shown to be effective in CRC treatment when used in combination with metformin and doxorubicin (167). This combination inhibits Hk, leading to a metabolic arrest with decreased ATP from glycolysis and reduced NADPH from the PPP. 2-DG has been observed to sensitize tumor cells to radiation and chemotherapeutic agents, increasing tumor control while sparing normal tissues (8). Systemic administration of 2-DG with focal tumor irradiation has direct effects on tumor cells, likely through changes in gene expression and phosphorylation of proteins involved in signaling, cell cycle control, DNA repair, and apoptosis (168). This treatment also promotes antitumor immunity in peripheral blood, with increased recruitment of innate and adaptive immune cells, a shift from Th2 and Th17 to Th1, and depletion of Tregs. Additionally, 2-DG combined with tumor irradiation has been shown to shift splenic macrophages towards an M1 phenotype, correlating with improved local tumor control (8). The inhibitor 6-aminonicotinamide (6-AN) targets the PPP by inhibiting G6PD, enhancing radiosensitization when combined with 2-DG (8). Treatments affecting the PI3K/Akt/mTOR and AMPK signaling pathways, such as 2-DG, have been found to increase memory T-cell presence by inhibiting glycolysis, thereby enhancing CD8+ T cell-mediated antitumor effects through increased AMPK activity and negative regulation of mTOR and Foxo1 (169). Metformin treatment also boosts AMPK activation and memory T-cell generation (170), potentially through its impact on mTOR signaling (171) or upregulation of miR33a, which reduces c-Myc expression (172). The secondary metabolite caulerpin has shown anticancer properties by disrupting the glycolytic process via the AMPK pathway (8). In the highly acidic TME, inhibitors of LDHA or MCT1 have been suggested to overcome resistance to ICIs in murine models. HIF-1α signaling, which controls several genes involved in glucose and lactate transport and glycolysis, such as GLUT-1, MCT1, and MCT4, is a key regulatory mechanism in this context (8). Quercetin, a natural compound and nonspecific MCT inhibitor, has shown significant effects in inhibiting proliferation, inducing cell death, reducing glycolytic activity, and enhancing the cytotoxicity of 5-fluorouracil (5-FU) in CRC cells (173). Other metabolic analogs, such as 3-bromopyruvate (3-BP) and lonidamine, target glycolysis by interfering with the activity of Hk. 3-BP also reduces MCT1 expression (174), while lonidamine inhibits it, thereby suppressing tumor progression (175). Carbonic anhydrases (CAs), which facilitate the conversion of water and CO_2_ to intracellular bicarbonate and a proton, work alongside MCTs in tumor cells to export excess protons and lactate, maintaining acid–base balance. Indisulam/E7070, a multiple CAs inhibitor (176) showing anti-tumor activity in xenograft CRC models, synergized when given in association with capecitabine or irinotecan in metastatic CRC patients (177). HIF-1 is crucial for sustaining high glycolysis rates in cancer cells. Bortezomib, a proteasome inhibitor that can inhibit HIF-1α transactivation, and topoisomerase I (TOP1) inhibitors like irinotecan/CPT-11 and EZN-2208 (a derivative of SN38, the active metabolite of irinotecan) that suppress HIF-1α/HIF-2α expression and HIF-induced targets are under clinical evaluation. However, in a Phase II trial (NCT00931840) involving patients with advanced CRC, EZN-2208, did not induce objective radiographic responses in KRAS-mutant patients resistant to CPT-11, with similar outcomes observed between cetuximab+EZN-2208 and cetuximab+CPT-11 groups, potentially due to unfavorable pharmacokinetics of EZN-2208 (177). Highly glycolytic tumors can deplete glucose and release large amounts of LA, inducing PD-1 overexpression and increasing the suppressive activity of Treg cells, partially explaining the limited efficacy of PD-1 blockade therapy (262). Treg cells actively uptake LA through MCT1, promoting NFAT1 translocation to the nucleus and enhancing PD-1 expression, while effector T-cell PD-1 expression is reduced. As a result, PD-1 blockade may inadvertently strengthen PD-1-expressing Treg cells, leading to treatment failure. Microsatellite-stable (MSS) CRCs, unlike MSI-H CRCs, exhibit lower responsiveness to ICIs, likely due to the Warburg effect contributing to an immunosuppressive TME and the induction of immune checkpoints. Combining glycolysis inhibitors with ICIs could potentially reverse resistance in patients treated with single-agent ICI (265, 268). In mouse models of CT26 CRCs, a combination of aspirin and anti-PD-1 therapy led to tumor growth reduction and complete response, outperforming monotherapy (178). Additionally, biological drugs like apatinib (a VEGF inhibitor) and trametinib (a MEK inhibitor) that can inhibit glycolysis, when used in conjunction with ICIs, may enhance therapeutic responses in CRC. Thus, emerging research supports the therapeutic benefits of combining glycolysis inhibitors with ICIs (179, 180).

### Targeting ICIs and/or lipid metabolism

Upregulation of lipogenic enzymes is commonly observed in patients with aggressive metastatic CRC, and targeting these enzymes, particularly FASN, a key element in the *de novo* biosynthesis of long-chain fatty acids, presents a viable therapeutic option. Cerulenin, the first FASN inhibitor, was found to decrease proliferation in murine CRC cells, promote apoptosis, and suppress liver metastasis of CRC (182). Moreover, when combined with oxaliplatin, it improved the efficacy of the treatment while reducing side effects (183). Luteolin, a potent FASN inhibitor with anti-tumor activity in CRC, likely operates by interfering with various signaling pathways, including the IGF-1 and Wnt-beta-catenin pathways (184). Epigallocatechin-3-gallate (EGCG), a polyphenol found in green tea, is another FASN inhibitor that has been shown to suppress proliferation and diffusion of CRC cells through STAT3 downregulation, significantly decreasing liver metastasis in SCID mice (181). Additionally, the acetyl-CoA carboxylase inhibitor (ACC) TOFA induced apoptosis in CRC cells (185). Targeting lipid metabolism alongside PD-1 inhibitors represents a novel therapeutic strategy to enhance the effectiveness of ICI blockade. Tregs are among the most significant intratumor immunosuppressive cells, and inhibiting lipid metabolism in Tregs is a focus of ongoing research. However, selectively targeting Tregs in tumors without harming cytotoxic T cells or triggering autoimmune diseases remains a major challenge. CD36, overexpressed on both Tregs and cytotoxic T cells, when deleted, decreased the expression of several immune regulatory receptors but not PD-1. Thus, inhibiting CD36 in intra-tumor Tregs through genetic ablation or using CD36 monoclonal antibodies effectively delayed tumor growth and enhanced the efficacy of anti-PD-1 treatment (26). Further studies on CD36 revealed that its overexpression in intra-tumor effector T cells exposes them to oxidative damage and ferroptosis due to lipid accumulation, and combining anti-PD-1 therapy with CD36 or ferroptosis inhibitors offered greater immunotherapeutic benefits (57). Senescent cytotoxic T cells also contribute to tumor immune evasion. Senescent and dysfunctional T cells exhibit aberrant lipid metabolism and intracellular lipid accumulation, with overexpression of group IVA phospholipase A2 (cPLA2-a) closely related to aging and lipid metabolism reprogramming in T cells via MAPK and STAT signaling pathways. Inhibiting cPLA2a prevented T-cell senescence and enhanced the efficacy of immunotherapies, such as adoptive T-cell transfer therapy (186). Moreover, activators of FAO, like bezafibrate, could potentiate anti-PD-1 therapy by inducing the expression of CXCL9 and CXCL10 from tumor cells and CXCR3 on intra-tumor effector T cells (187, 188). In a recent *in vivo* study (189), the expression of chemokine ligand 10 (CXCL10) was linked to enhanced CD8+ T-cell infiltration, and upregulation of CXCL10 improved the responsiveness of CRC cells to a combined treatment of cetuximab and anti-PD-1, unlike when these treatments were administered individually. The relationship between MDSCs and resistance to ICB appears to hinge on differing gene expressions of CD8+ T cells between sensitive and resistant patients. Inhibiting the function of PIM1, a serine/threonine kinase, through pharmacological means or genetic ablation reduces FA uptake and FAO in MDSCs via a PPARgamma-mediated pathway, leading to a significant decrease in MDSCs, increased recruitment of CD8+ T cells, and improved resistance to ICB (190).

### Future directions

“Glycolytic enzymes,” traditionally considered “housekeeping” proteins, have been identified as “moonlighting” proteins that perform multiple functions. These roles are influenced not just by their structural peculiarities but also by compartmentalization and the metabolic environment (269). The interplay between cell metabolism and gene transcription provides specific molecular and functional targets for cancer treatment, with many enzymes catalyzing reactions in the glycolytic pathway to lactate production also involved in regulating gene transcription (270). Understanding the dual roles of glycolytic enzymes in gene expression regulation within tumor cells offers a novel approach to cancer treatment, targeting the additional functions of these metabolic enzymes. However, designing pharmaceutical inhibitors that specifically target these moonlighting functions remains challenging, and the potential of such inhibitors in cancer therapy is yet to be fully determined. Ferroptosis, an iron-dependent programmed cell death induced by lipid peroxide accumulation and ROS, represents another emerging area for cancer treatment strategies (271). Experimentation has shown that manipulating ferroptosis in tumor cells can enhance the effectiveness of anti-PD1 therapy (8). AZD3965, an MCT1 inhibitor, is currently being investigated in clinical trial NCT01791595 (262). Moreover, elevated serum ammonia levels have been observed in CRC patients (272), with an ammonia-related gene signature correlating with poorer prognosis and lack of response to ICIs, suggesting that targeting tumor-associated ammonia could enhance the efficacy of immunotherapy, including PD-L1 inhibitors. Nanocarriers is further promising therapeutic strategy for drugs targeting the PD-1/PD-L1 pathway. In fact, nanocarriers represent a rationally conceived intelligent delivery system that can control therapeutic agent delivery and improve tumor targeting ability. Nanocarriers are responsive to tumor acidic microenvironment, high level of GSH and ROS, and specifically upregulated enzymes (internal stimuli) or light, ultrasound, and radiation (external stimuli). Therefore, they can carry out the target immunomodulators at the tumor site, increasing anti-tumor efficacy but reducing off-target toxicity (273). Lastly, abnormal microbial metabolites and microbial dysbiosis significantly impact CRC pathogenesis. Therefore, improving gut microbiota through dietary interventions, probiotic/prebiotic supplementation, or the administration of beneficial microbial metabolites could offer an additional therapeutic strategy for CRC.

## Discussion

The Warburg effect, recognized as a key player in the metabolic reprogramming of cancer cells, is among the 10 widely accepted hallmarks of cancer. Nonetheless, emerging evidence suggests that tumor growth may also rely on mitochondrial metabolism, to some extent. In the TME, proliferating tumor cells exhibit shared metabolic reprogramming pathways to meet increased nutrient and oxygen demands. Hypoxia in the TME often leads to a metabolic shift from OXPHOS to aerobic glycolysis, followed by enhanced lipid synthesis and glutaminolysis due to lactate acidification. This acidification provides cancer cells with a growth advantage over immune cells, inhibiting T-cell proliferation, promoting the migration of neutrophils and DCs, and increasing Tregs and the infiltration of MDSCs and TAMs, thereby fostering an immunosuppressive TME. High lactate levels suppress T-cell proliferation and IFN-gamma release, while low LDHA expression in T cells promotes their conversion to FoxP3+ Tregs, further encouraging PD-1 expression. Nutrient deficiency in the TME leads to the polarization of M1 macrophages to immunosuppressive M2 TAMs, while certain chemokines attract MDSCs to the TME, where they secrete immunosuppressive cytokines. Hypoxic conditions promote the immune-metabolic reprogramming of MDSCs towards FAO, with AMPK activation and FAO enhancing their immunosuppressive activity. Overexpression of AMPK in TADCs and increased abnormal lipids convert them into tolerogenic DCs. High levels of TGF-beta, coupled with decreased OXPHOS, inhibit NK cell function, while high lactate levels induce mitochondrial dysfunction, increased ROS and apoptosis in NK cells. In CRC, studies have documented abnormal glycolysis, glutaminolysis, and enhanced lipid synthesis, including high lactate levels and upregulation of GLUT1, Hk2, and enzymes involved in glycolysis, as well as ASCT2 upregulation. High lipid levels and increased lipogenesis, along with altered FA metabolic pathways in CRC, have been linked to tumor growth and poorer outcomes. Acidosis in CRC cells impairs the activity of anti-tumor immune cells, inhibits IFN-γ production by CD8+ T cells, and suppresses TAM phagocytosis. The competitive nature of nutrient availability in the TME promotes the formation of immunosuppressive phenotypes such as Tregs and M2-like macrophages. GPR81 signaling activation by lactate in CRC cells induces PD-L1 overexpression and decreases the pro-tumor capability of TAMs. Lactate can also activate GPR81 in DCs, diminishing their antigen presentation capabilities. The genetic and epigenetic phenotype of cells, along with the microbiota, plays a significant role in the metabolic reprogramming of CRC. High acidity and lactate levels in the TME, through various mechanisms, inhibit T-effector cell activities, while certain bacteria increase SCFA production, enhancing CD8+ T-cell activation and inducing genes involved in antigen processing or presentation. However, other microbiota mechanisms may promote pro-tumor activities. Reports of successful outcomes with ICIs in selected CRC patients have emerged, and the combination of these immune therapies with drugs targeting metabolic pathways, particularly glycolysis and lipolysis, is being extensively investigated to enhance clinical outcomes and expand the population benefiting from immunotherapy.

## Author contributions

AN: Writing – original draft, Writing – review & editing. PF: Writing – original draft, Writing – review & editing.
